# Radar human breathing dataset for applications of ambient assisted living and search and rescue operations

**DOI:** 10.1016/j.dib.2023.109757

**Published:** 2023-11-10

**Authors:** Cansu Eren, Saeid Karamzadeh, Mesut Kartal

**Affiliations:** a Satellite Communication and Remote Sensing, Department of Communication Systems, Informatics Institute, Istanbul Technical University, Istanbul, Türkiye; b Millimeter Wave Technologies, Intelligent Wireless System, Silicon Austria Labs (SAL), 4040 Linz, Austria. Electrical and Electronics Engineering Department, Faculty of Engineering and Natural Sciences, Bahçeşehir University, 34349 Istanbul, Türkiye; c Department of Electronics and Communication Engineering, Istanbul Technical University, Istanbul, Türkiye

**Keywords:** Ultrawideband (UWB), Vital signs, Ambient assisted living, radar, Monitoring

## Abstract

This dataset consists of signatures of human vital signs that are recorded by ultrawideband radar and lidar sensors. The data acquisition scene considers the human posture models(supine/lateral/facedown), different radar antenna angles towards the human, various set of distances and operational radar characteristics (bandwidth selection/mean power). The raw data files of lidar&radar and processed data files are presented separately in the data repository. The lidar sensor is chosen as a reference sensor. There are 432 data records, and each data scene's trial number is eight. There is a homogeneous wooden table to mimic clutter while forming a dataset. Thus, this dataset covers applications of search and rescue operations, sleep monitoring, and ambient assisted living (AAL) applications.

Specifications Table

**Table:** 

Subject	Electrical and Electronic Engineering, Signal Processing, Data Science
Specific subject area	Non-contact monitoring of human vital signs; through wall UWB radar; ambient-assisted living (AAL); artificial intelligence& machine learning
Type of data	Radar Data (Calibration, Processed, Raw Radar, ErrorAnalysis) and Reference Data (Lidar) are given in “. mat" format. The codes are provided as “. m." A brief explanation for the data scene “.pdf,”, and the licenses “.txt” for coding are also included.
How the data were acquired	The proposed dataset is formed by FlatEarth's UWB Radar Solutions: Salsa Ancho radar module and ST life. augmented: VL53L0X laser-ranging sensor (Lidar). XETHRU X2-Impulse Radar Transceiver by NOVELDA, transmitting and receiving antennas are placed in BeagleBone Black cape, which is the element of the Salsa Ancho radar module that enables the data transmission. The data recording and the communication interference between the Salsa Ancho radar module and computer is established using SalsaLab MATLAB Toolbox. The data recording and communication interference between Lidar sensor and computer is established using Arduino IDE and MATLAB.
Data format	Raw (Radar, Calibration, Reference), Processed Data and Error Calculation
Description of data collection	The proposed data is recorded at 1.75 GHz,2.5 GHz, and 3.1 GHz bandwidths. The threshold signal value of the bandwidth is at −10 dB. The clutter is chosen as a 3-cm thick wooden table. The UWB radar sensor is placed above the wooden table and this distance is varied between at 8 cm to 27 cm, and the lidar sensor is located under the wooden table. Supine, lateral, and face-down human orientations are regarded through the UWB radar and lidar device. The trial number of data recordings for each data scenario was 8. The duration of each data recording is 58.6 s. The radar orientations are chosen at angles where 0° and 30° towards the human chest. The distances between radar and lidar sensors and human is varied between 46 cm to 78 cm. The human is 32 years old, and the height&weight of the human is 173 cm, 59 kg. The size of the human chest is 30cmx23cm. While data collection, the human is lying steadily on the floor. While data recording, the lidar and radar sensors are placed so that the observation of the human chest is clear. The reference and radar data recordings are realized simultaneously. After raw data collection, the processed data is formed to extract breath frequencies. The calibration data is acquired where the human subject is absent in the data scene.
Data source location	• City: Maltepe/Istanbul • Country: Turkey
Data accessibility	Repository name: Mendeley Data Data identification number: 10.17632/cbj37wdsdj.2 Direct link to Data

## 1 Value of the Data

• This dataset allows researchers to analyze and develop applications for non-contact monitoring of vital signs, ambient assistant living (AAL) and search rescue operations after disasters.
• This dataset provides a brief data source for artificial intelligence applications that automatize and enhance vital human sign detection in various fields.
• This dataset contributes literature to examine near antenna field regions and cluttered environments.
• Enabling usage of the selected set of primary parameters during data acquisition, that is, the type of human posture, operational radar characteristics, the cluttered environment, different sets of ranges and radar positions, the dataset provides better understanding and visualization to researchers.

## 2 Objective

The developments in ultrawideband (UWB) radar technology validate the capability of detection&monitoring of vital human signs via radar techniques. The proposed dataset aims to provide detailed human vital records regarding human posture orientation, selection of operational radar characteristics, radar positioning through human body, different set of ranges and near-field clutter environment.

## 3 Data Description

Contactless monitoring of human vital signs is an appealing technology in medicine, defense, and search and rescue operations [1]. The growing rates of the aging population in the world and the past COVID pandemic disclose the need for remote devices that can examine the physical activity of human beings at their houses, diagnose illnesses, and enhance the life quality of older adults [2,3]. Blood pressure, body temperature, breath, and heart rate are the principal vital signs of human-beings [4]. The breath rate indicates the vividness of the human body and causes more enormous distance changes in the human chest compared to the heartbeat motion [5]. Thus, the detection of the breath is crucial for vital sign monitoring. The breath rate is detectable using contact-based and remote sensors such as electrocardiography, strain-based sensors, photoplethysmography, and radar systems [4]. The standard breath frequencies of human beings are 0.2–0.33 Hz [6]. Gender, age, weight, abnormalities in lung expansion and contraction while breathing, and exercise affect the breath rates of humans. Thus, breath rate estimation is applied in rigorous biomedical applications such as anomaly detection(obstructive sleep apnea, sudden infant death syndrome) [2,^7^,8]. The other main research fields of human vital sign monitoring are the physical examination of athletes during exercise search and rescue of humans after natural disasters such as earthquakes and avalanches [9].

Contact-based sensors require attachment to the human body to monitor breath rates that cause skin irritations for more extended observations. Contact-based sensors are prone to motion artifacts and can not be used on injured tissue. Thus, such sensor selection reduces patient comfort in hospitals and houses. Moreover, these systems are invalid in search operations after natural disasters [10].

UWB radar systems offer a high-range resolution, non-ionized, contactless, and rapid data acquisition [11].Regarding their higher bandwidth solutions, tiny movements of the human chest while breathing are sensible by UWB radar systems [12].. The other radar systems can remotely detect human vital signs, such as continuous wave radars, frequency-modulated continuous wave radars, non-linear radars, and stepped-frequency continuous-wave (SFCW) Radars [13,14]. Machine learning algorithms promote the accuracy of human vital signs, such as breath rate in various fields, making radar systems a great candidate for monitoring human vital signs in varios data scenarios. The challenges of breath rate detection via radar systems are the human orientation towards the radar sensors, cluttered environment, random body movement (RBM), the orientation of radar systems, movement of humans while data collection, and multiple human-beings in the data records [15,16]. The selection of the operational bandwidth/center frequency and the transmitted power can enhance the accuracy of the breath rate estimation via radar systems. However, there still needs to be more public shared radar vital sign datasets to test the ability of the current machine learning algorithms. In this proposed dataset, we take into consideration the human postures, different sets of antenna positions, the various settings of ranges, and operational radar characteristics such as bandwidth selection.

**Fig. 1:**
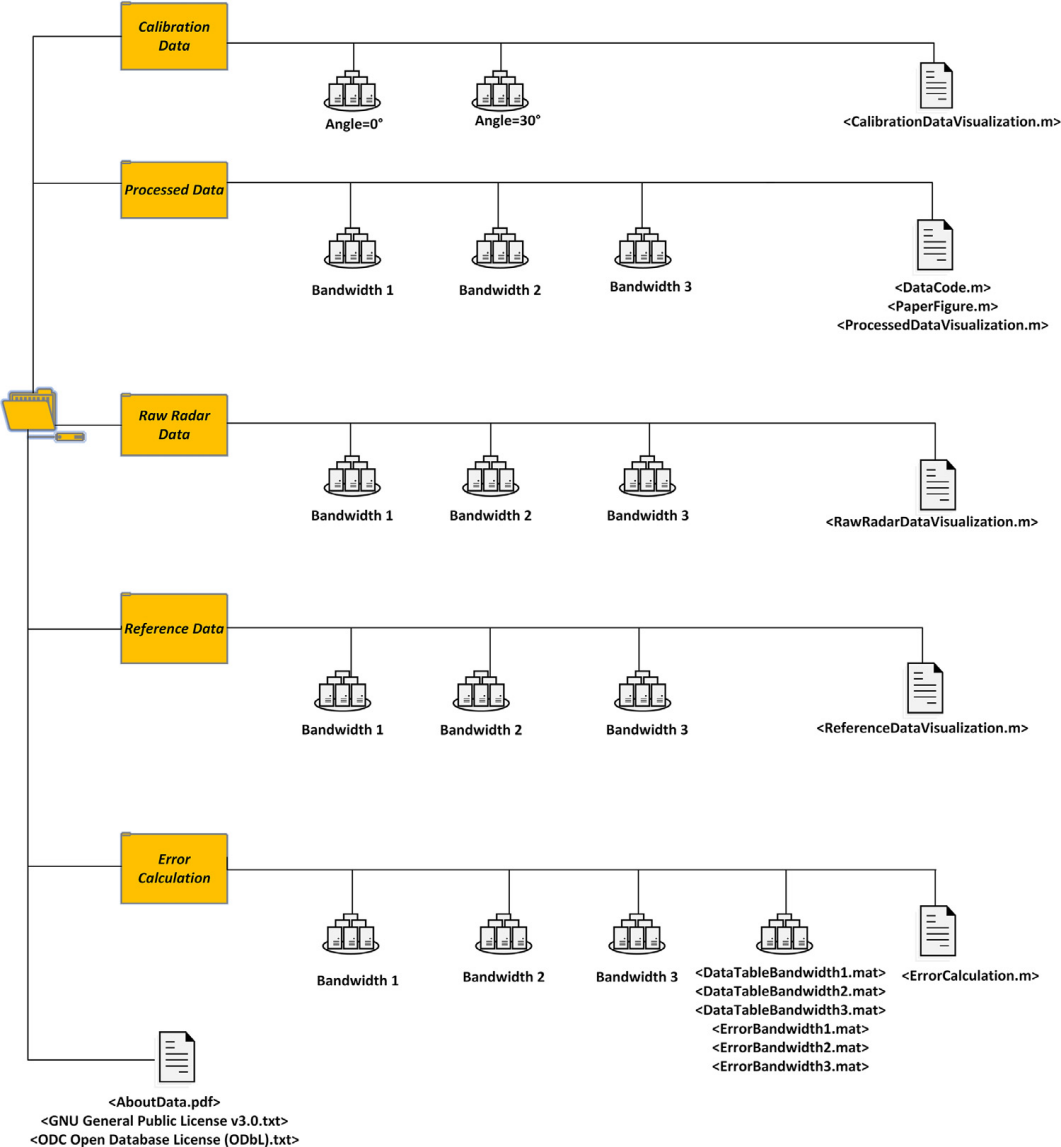
The proposed data file structure.

The file structure of the proposed data is given in Fig. 1. The data file consists of sub data files “Calibration Data,” “Processed Data,” “Raw Radar Data,”, “ErrorCalculation” and “Reference Data”. There is additional documentation for the data, such as licenses for coding files&database, a brief explanation of data as “AboutData.pdf” Each data file is consisted of sub-files such as bandwidth1, bandwidth2, and bandwidth3, except “Calibration Data”. Each sub-file covers two different sets of angle orientation of radar systems, three different types of human posture, and the six different sets of range differences for “Processed Data,” “Raw Radar Data,” and “Reference Data.” The “ErrorCalculation” data file is the place for where the relative absolute error calculations are made for each bandwidth selection. “Calibration Data” is the experiments performed where the human is absent. After applying the techniques given in “Section: Experimental design, materials and methods”, the data is recorded in “Processed Data”.

Processed data consists of the background-subtracted data (“BS_…”), estimated breath radar (“FilteredBreathRadar_…”), reference signals (“FilteredBreath_Ref_…”), the spectrum of breath&reference signals (“SpectrumRadar_…”, “SpectrumRef_…”), estimated radar and reference breath frequencies matrixes (“EstimatedBreathFrequencyRadar.mat”, “EstimatedBreathFrequencyRef.mat”). Moreover, signal-to-noise ratio calculations of UWB radar data and reference data are included in “Processed Data” as “RefSignalToNoise.mat” and “RadarSignalToNoise.mat”. The histogram presentation of the absolute relative error calculations and error presentations are given as “Error#.fig” and “Histogram#.fig.” Human posture in the data naming refers to the subject orientation for supine/lateral/facedown. The operational radar bandwidth name indices are “…Band# (1,2,3)” …DeltaR=#(27 cm, 22.5 cm, 20 cm, 15.5 cm, 10 cm and 8 cm)… “refers to the distance between UWB radar and the wooden table.” …Angle=#(30° and 0°…) “presents the UWB radar's orientation through the human chest. The repeat number of the data recording is given by “…Trial#”.

The local error data of each data record scene and the error data of bandwidth selections are given in the “ErrorCalculation” data file as “ErrorBandwidth#.mat” and “LocalErrorTable#.mat.” We also include the “DataTableBandwidth#.mat” that is consisted of estimated breath frequencies and signal-to-noise ratios.

**Fig. 2:**
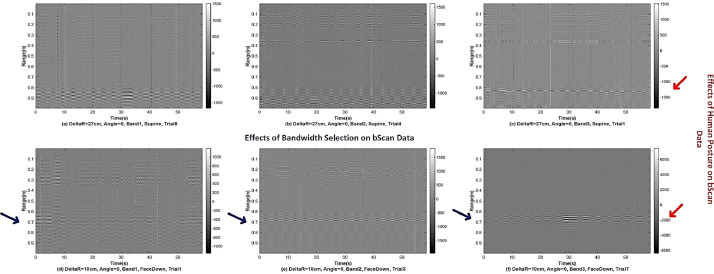
The UWB radar data record samples that regard bandwidth selection and human posture.

**Fig. 3:**
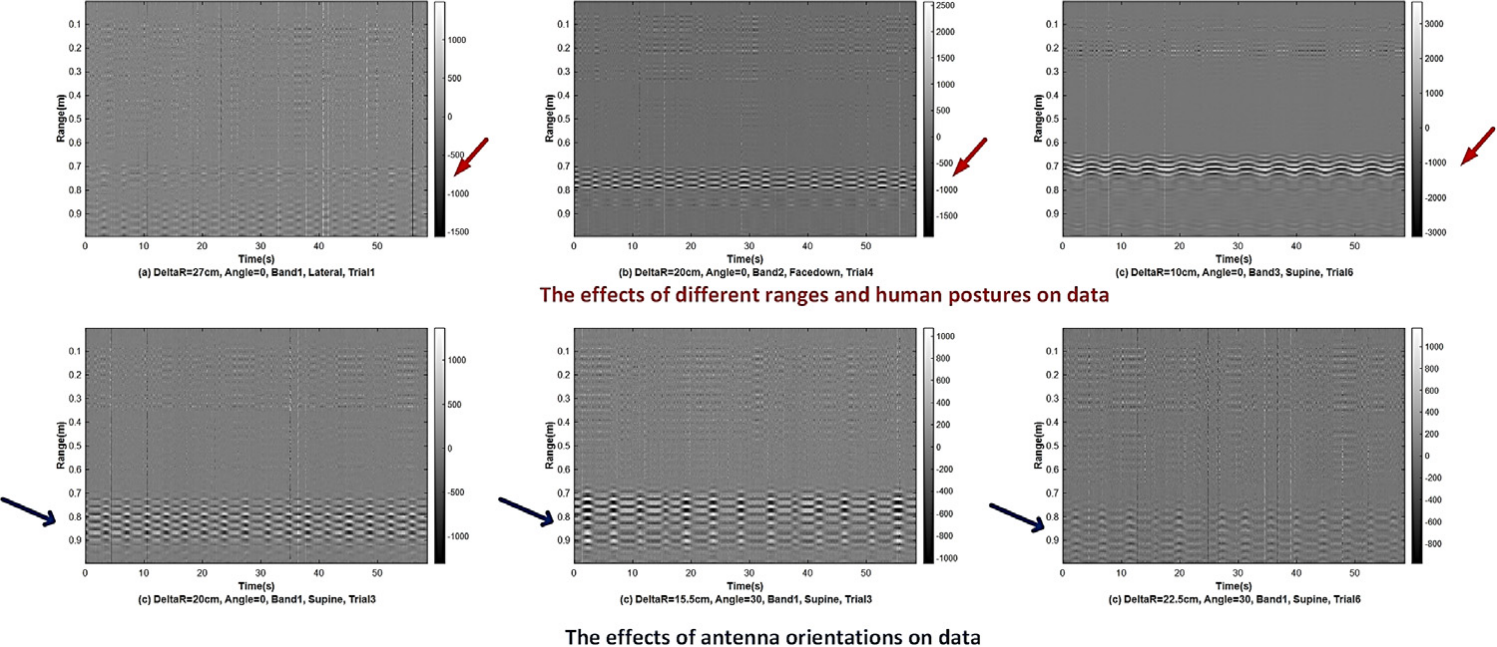
The UWB radar data record samples that regard different ranges and antenna orientations.

Fig. 2 shows the bScan radar data records of different set bandwidth selection and human postures. The x-axis shows the time duration for the data collection, which is slow time, and the y-axis refers to the range in meters. Fig. 3 presents the data records where the human is located at different ranges and the UWB radar is positioned at 0° and 30°. The time duration for each data is 58.6 s and the unambiguous range is up to 1 m.

**Fig. 4:**
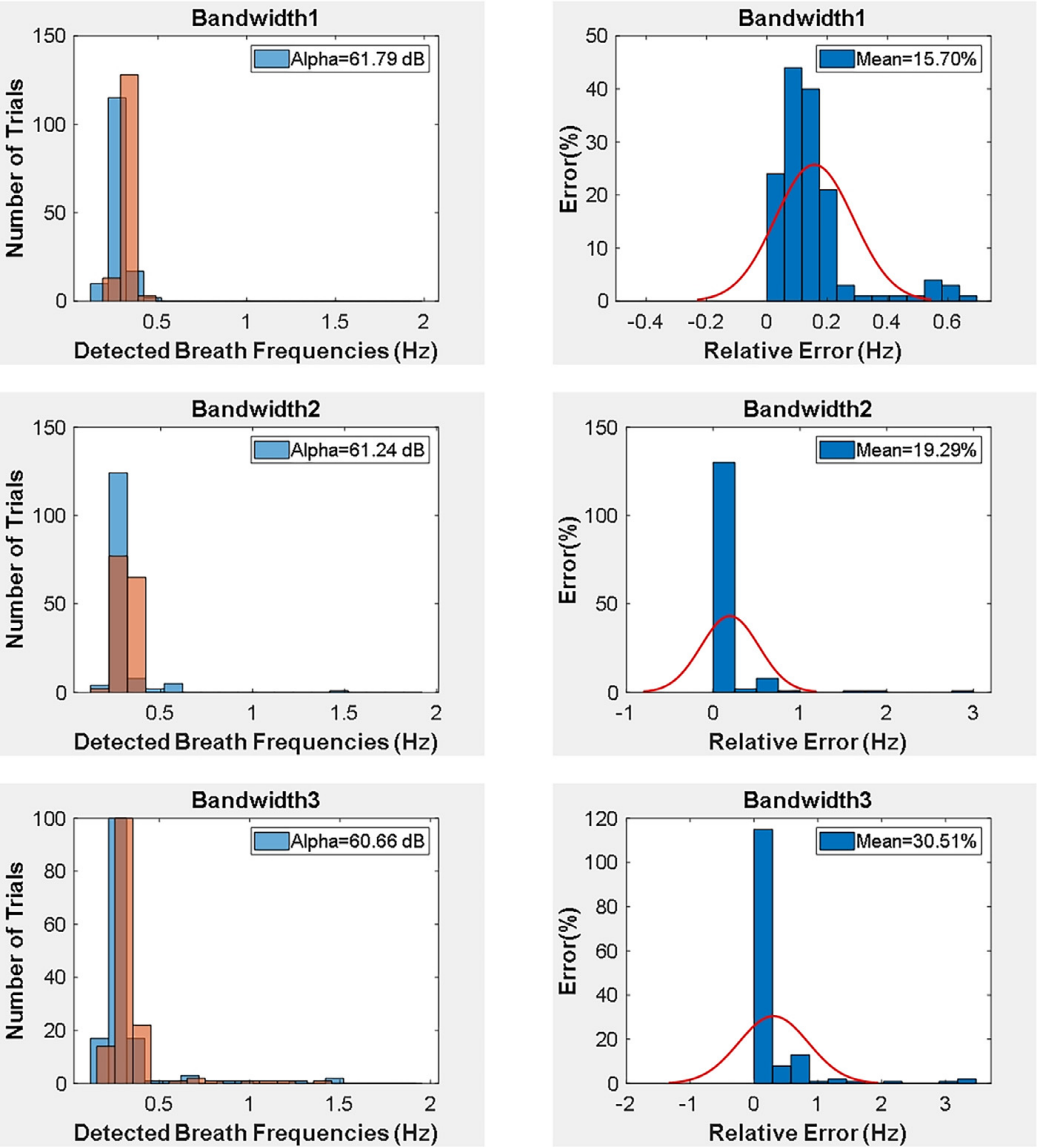
The density representation of detected breath frequencies and the absolute relative error rates of UWB breath data.

The histogram and absolute relative error of data that considers bandwidth selections are given in Fig. 4. The absolute relative error is calculated based on the formula in (1.1) where $f_{\text{radar}}$ is the estimated breath frequency and $f_{\text{reference}}$ is the estimated breath frequency of lidar sensor. N represents the number of sets of data trials. The alpha corresponds to signal to ratio values of each bandwidth selection on histogram data in Fig. 4.

(1.1) $$ɛ=100x\;\frac{\sum_{n=1}^{N}\frac{\left|f_{\text{radar}}-f_{\text{reference}}\right|}{f_{\text{reference}}}}{N}$$

## 4 Experimental Design, Materials and Methods

This section explains the experimental design of data collection and the methodology used to extract “Processed Data”.

### 4.1 Experimental design

**Fig. 5:**
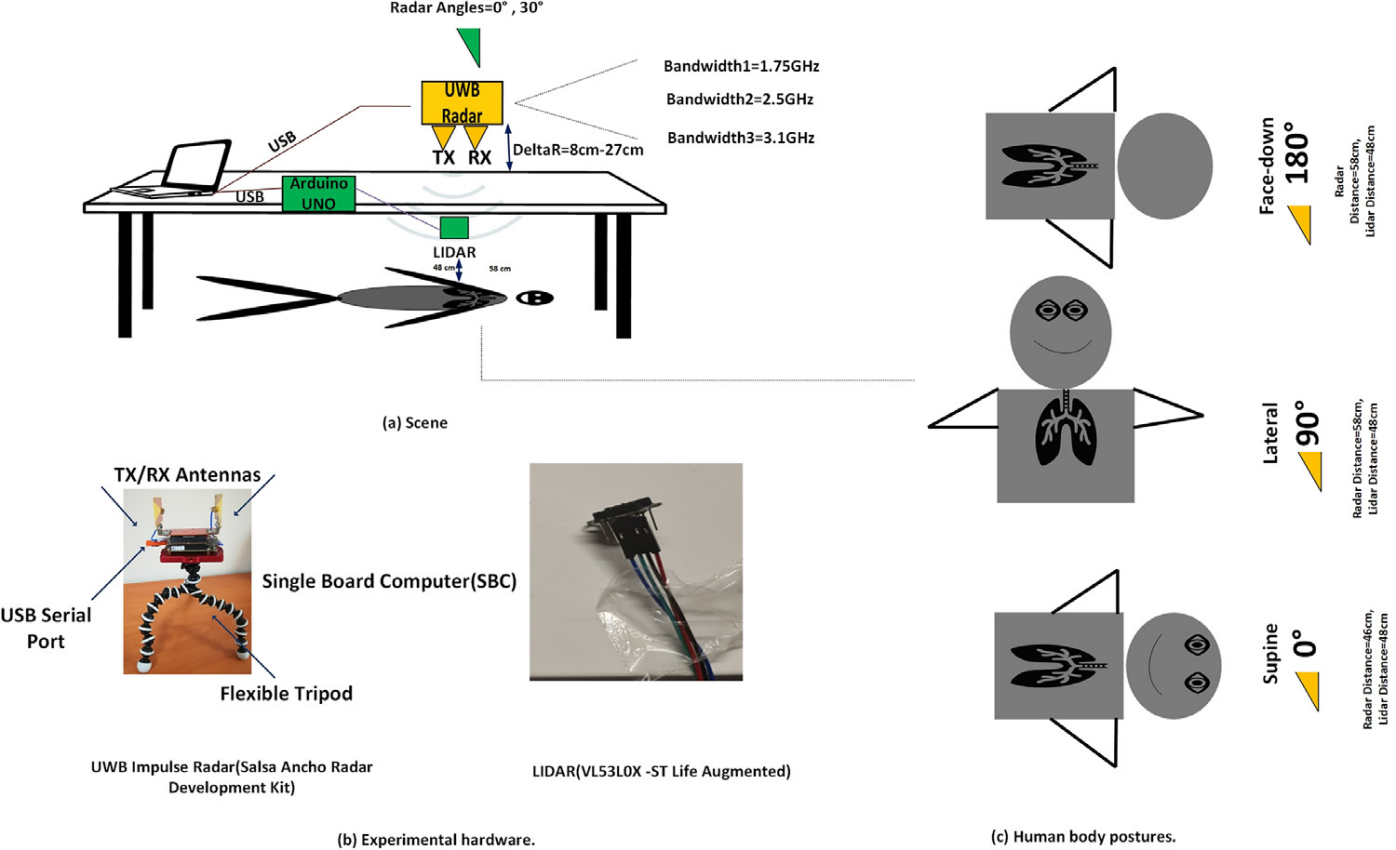
Experimental design. (a) Depiction of experimental scene regarding selection of radar operational parameters, antenna and subject orientations. (b) The experimental setup was used in dataset acquisition (c) Human body postures*.*

The model for the TWR data collection and its types of equipment is shown in Fig. 5(a)-(b). Salsa Ancho Radar Module and STlife.augmented range and gesture sensor are used during data collection [17,18]. Both systems are applicable to biomedical applications and provide safety considerations for human experiments. The proposed radar system operates on 4.5GHz- 9.5 GHz. The bandwidth of the radar system is adjusted as 1.75 GHz,2.5 GHz and 3.1 GHz at −10 dB using graphical interference of the equipment. The center frequencies are 5.3 GHz,7.7 GHz and 8.8 GHz. The mean power at that region are −10.7 dBm, −14 dBm, and −16.4 dBm, respectively. The range accuracy of the radar is 4 mm and up to 1.2 mm for the lidar sensor. The dimensions of the radar system and lidar sensors are 58.42×54.61 mm and 4.4 × 2.4 × 1 mm. The antenna dimensions of radar sensor are 4.2cmx6.5 cm. The radar geometry is monostatic. MATLAB 2020A and Arduino IDE are used during the acquisition of radar and lidar data.

A homogeneous 3-cm thick wooden table is positioned between the human and UWB radar sensor. The distance between the radar and the table was adjustable and varied between 8 cm to 27 cm. The range between human and lidar was 48 cm, and the lidar was placed under the wooden table where it directly saw the human chest.

The selection of clutter type is made due to simple and well-known characteristics of the wood. The distances between human and radar systems vary depending on the subject's posture. The human was lying down steadily for 58.57 s. The data recordings were repeated eight times in each scenario. The human subject orientations were organized as supine/lateral/facedown. The range resolution of the UWB radar were 8.6 cm, 6 cm and 4.8 cm.

The radar system was positioned at two different angles during data recording. The data record parameters are given in Table 1. The corresponding posture definitions and their range between human-radar/human-lidar is shown in Fig. 5(c).

**Table 1:** The parameters of data record and scenarios.

Human Body Position	The Radar Center Frequency	The Radar Mean Power	The Radar Bandwidth	Distance-Between Target-Radar	Angle Variations of Radar	Distance Between Target-Lidar	Number of Trials
Face-Down, Supine, Lateral	5.3 GHz, 7.7 GHz 8.8GHz	−10.7 dBm, −14 dBm −16.4dBm	1.75 GHz, 2.5 GHz and 3.1GHz	59cm-78cm	00, 300	48cm	8

The radar and lidar sensors work together simultaneously. Fundamentally, the proposed radar system emits higher-order Gaussian modulated signals through the data scene using transmitter antenna. The emitted radio waves are reflected from the table, human chest, and surrounding objects. Each object is resulted a time delay in echo signal and collected by the receiver. After the sampling process, the radar data is formed. Unlike radar system, the lidar transmitter sends the light waves within 940 nm wavelength through the scene. The emitted light waves bounce back from the scene and collected by receiver. The lidar sensor records the ranging value of the targets. Regarding periodic movement of human chest while breathing, the data ranging values are varied. Thus, the recorded radar and lidar ranging values are closer while the lungs are inflated and far away at the deflated phase of the lungs.

### 4.2 Methods

The schematic of the data recording scene has already given in Fig. 5. This section explains the mathematical description of the scene and the methodology that used while forming “Processed Data” file. The nominal distance ($d_{0})$ between human subject and UWB radar system is given in (1.2).

(1.2) $$d_{0}=∥X_{h}-X_{\text{tx}}∥+∥X_{\text{rx}}-X_{h}∥$$

where the positions of human, transmitter and receiver antennas are given as $X_{h}$, $X_{\text{tx}}$, and $X_{\text{rx}}$. Time-varying distance due to the movement of the chest while breathing is given regarding nominal distance offset in (1.3). The breath and heartbeat frequencies are $f_{b}$ and $f_{h}$ in (1.3). The slow time vector is symbolized by t. The amplitudes that correspond to breath and heartbeat movement are given by $m_{b}$ and $m_{h}.$The values of human breathing and heartbeat frequencies are 0.2–0.33 Hz, 1–1.33 Hz (3).

(1.3) $$d\left(t\right)=d_{0}+m_{b}\sin\left(2\pi f_{b}t\right)+m_{h}\sin\left(2\pi f_{h}t\right)$$

The time of arrival between human and the UWB radar system is given in (1.4).

(1.4) $$\tau_{d}\left(t\right)=2d\left(t\right)\;/\;c=\tau_{0}+\tau_{b}\sin\left(2\pi f_{b}t\right)+\tau_{h}\sin\left(2\pi f_{h}t\right)$$

where c is the speed of light in vacuum, $\tau_{0}$ is the nominal delay time, $\tau_{b}$ and $\tau_{h}$ are the breathing and heartbeat related time delays. The received radar breath signal is convolution of transmitting signal and impulse response of the system where τ is the fast time in (1.5). The impulse response of system is given by h(t,τ) and s(τ) is radar transmitting signal in (1.5).

(1.5) r(τ,t)=s(τ)*h(t,τ)

Since UWB radar collects the echo signal reflected from the environment, such as clutter, walls, and humans, the received data is the superposition of echoes corresponding to different delays in slow time. The received signal is given in (1.6) where $A_{n}$ is the amplitude of echo signal at nth sample of bScan radar data.

(1.6) $$r\left(t\right)=\sum_{n=1}^{N}A_{n}s\left(t-\tau_{n}\right)$$

**Fig. 6:**
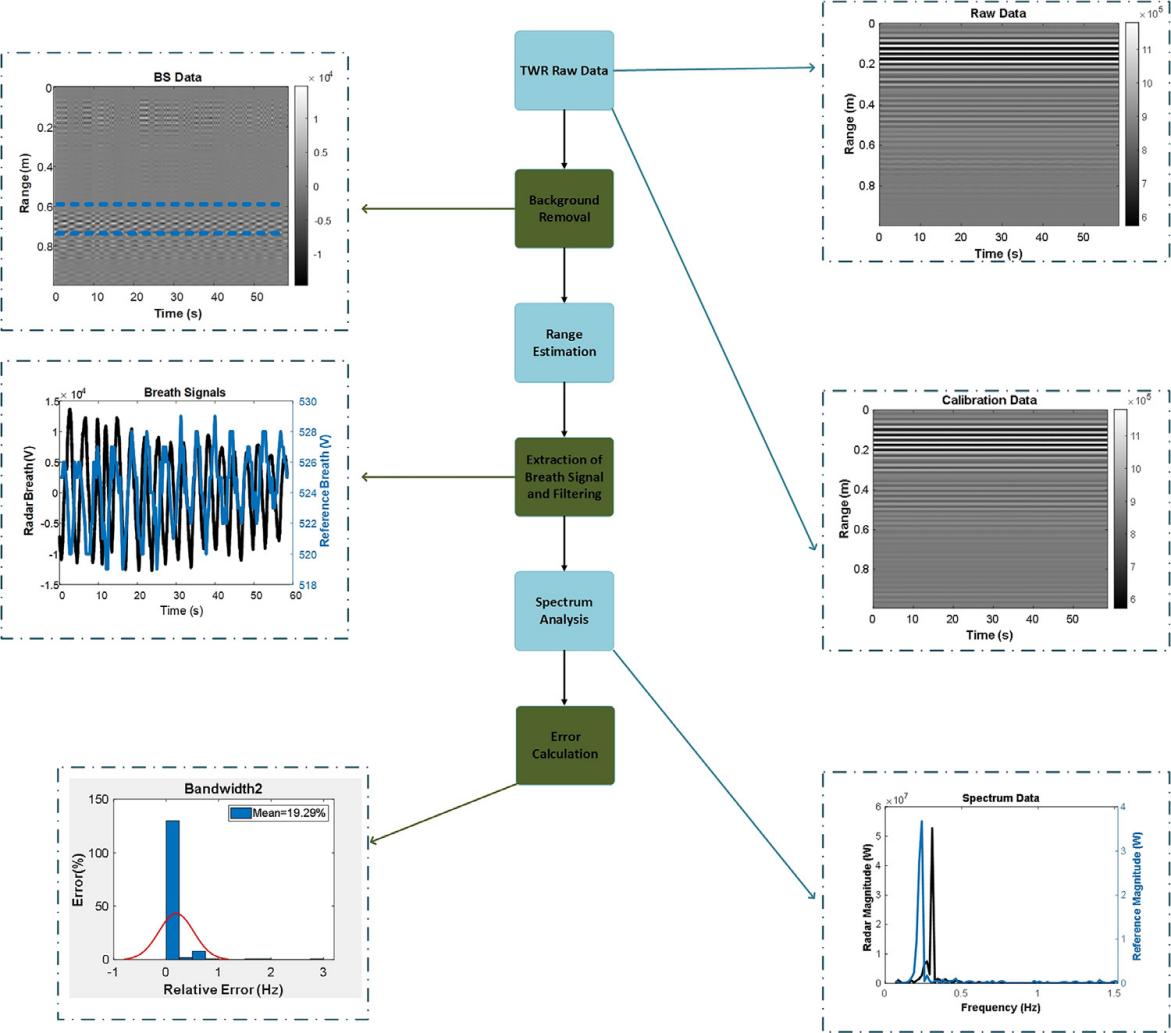
The flowchart of the proposed dataset, DeltaR=10cm_Angle=0_Band1_Trial1.

The received signal includes the clutter and noise. After sampling process, the received signal is presented as:

(1.7) R[m,n]=h[m,n]+c[m]+n[m,n]

where h[m,n] corresponds to the presence of human body, c[m] is clutter, n[m,n] is total noise where m is the sampled fast time and n is the sampled slow time index. An example of data processing of UWB radar breath signal and reference signal is given in Fig. 6. The background removal of the collected radar data is achieved by Linear Trend Subtraction. The signal without clutter is represented as in (1.8).

(1.8) $$R\left[m,n\right]=\alpha_{v}s\left(m\delta_{R}-v\tau_{v}\left(nT_{s}\right)\right)$$

where Ts is the pulse repetition interval of UWB radar, *t* = $nT_{s}$, *n* = 0,…,N-1, $\delta_{R}$ is the sampling interval in slow-time samples and v is the speed of light. The breath frequency can be extracted by Fourier Transform of (1.5) in slow time which is expressed in (1.9) [19].

(1.9) $$Y\left(m\delta_{T},f\right)=\int_{-\infty }^{\infty }Y\left(m\delta_{T},t\right)e^{-j2\pi \text{ft}}\text{dt}$$

Where $\delta_{T}$ samling interval in fast time, f corresponds to frequency and t is the time. After range estimation and extraction of the breath signal, we applied a low-pass filter to eliminate unwanted noise. Then, we estimated the breath frequencies of reference and radar data.

The radar dataset articles are published by researchers in the literature are given in Table 2. The reference equipment, radar type and their operational characteristics, data fields, data formats, and the number of trials are presented. The datasets are focused on human activity recognition that considers hand gestures, macro-human movements such as walking, falling, etc., and breath and heart rate estimation that regards apnea, post-exercise, speech, etc. The angle variations, human postures, and the cluttered environment are viewed in some cases, such as human-motion recognition. However, the datasets that focus on biomedical and civilian applications need to be shared to develop ambient-assistant living systems and search and rescue radar systems and enhance their accuracy. Thus, In our dataset, we take the operational radar characteristics, human postures, different ranges, and antenna orientations as data recording parameters.To validate our data, we used lidar as an additional source.

**Table 2:** Datataset research in the literature.

Ref.	Year	Reference Equipment	Radar Type	Radar Frequency	Methodology	Scenarios	Trial Number	Data Format
Proposed	2023	Lidar	UWB	5.3 GHz, 7.5 GHz, 8.8 GHz	FFT	Biomedical and Ambient-Assistant Living	8	.mat
[20]	2022	ECG and respiratory belts	Continuous Wave	24 GHz	FFT	Biomedical	1	.csv
*(5)*	2022	–	FMCW	5.8 GHz, 400 MHz Bandwidth	FFT and classification algorithms	Human Activity Recognition	Up to 3	.dat
[21]	2022	–	Continuous Wave	Human Activity Recognition	FFT, STFT and classification algorithms	Human Activity Recognition	–	.jpg, .mat
[22]	2020	PPG, ECG, PCG, radar, respiration sensor	Continuos Wave	24 GHz	R peaks and T-wave end detection, STFT	Biomedical	Up to 30	.mat and .csv
[23]	2022	Kinect sensor, Wifi Channel State Information	UWB	6.5 GHz	Convolutional neurol network based classifier	Human Activity Recognition	Up to 6	.mat and .csv
[24]	2021	–	UWB	500 MHz	FFT, STFT, EEMD, LTS Convolutional neural network	Human Activity Recognition	–	.jpg
[25]	2021	–	UWB	Biomedical research	Convolutional neurol network based classifier	Human Activity Recognition	1	.csv,.mat
[26]	2020	Task Force Monitor 3040i, ECG, ICG,	Continuous wave	24 GHz	Hidden-semi Markov model based segmentation, R-peak estimation	Biomedical	1	.mat

## Ethics Statements

This study was approved by the Ethics Committees on Istanbul Aydın University on 24.07.20217 with the report number:2017/13 and Bahçeşehir University on 11.12.2019-E.3051 with the report number: 20021704-604.02-.

## Funding

This study was supported by Istanbul Aydın University, Scientific Research Project (BAP) research fund in 2017.

## CRediT authorship contribution statement

**Cansu Eren:** Data curation, Formal analysis, Investigation, Methodology, Software, Validation, Visualization, Writing – original draft. **Saeid Karamzadeh:** Supervision. **Mesut Kartal:** Supervision.

## Data Availability

Radar Human Breathing Dataset for Applications of Ambient Assisted Living and Search and Rescue Operations (Original data) (Mendeley Data)
